# Diet and Dementia: A Prospective Study

**DOI:** 10.3390/nu13124500

**Published:** 2021-12-16

**Authors:** Hikaru Takeuchi, Ryuta Kawashima

**Affiliations:** 1 Division of Developmental Cognitive Neuroscience, Institute of Development, Aging and Cancer, Tohoku University, Sendai 980-8575, Japan; ryuta@tohoku.ac.jp; 2 Smart Aging Research Center, Tohoku University, Sendai 980-8575, Japan; 3 Department of Advanced Brain Science, Institute of Development, Aging and Cancer, Tohoku University, Sendai 980-8575, Japan

**Keywords:** diet, dementia, bread, meat, fish, vegetables, fruit

## Abstract

Whether dietary and nutrition and dietary patterns are associated with the development of dementia is an interesting research question. Participants of a longitudinal cohort study that included European adults who were middle to old aged at baseline and who had not been diagnosed with dementia at baseline (2006–2010) and had not been diagnosed with dementia or died within 5 years after baseline were followed up (until 2018) and analyzed. Associations between intake frequency of each food class measured by the food-frequency questionnaire at baseline and incident dementia 5 years after baseline were analyzed after correcting for confounding variables. A total of approximately 340,000 participants and 900 cases were included in the analysis for each food class. Cox proportional hazard models with self-reported intake level of each food category divided into four mostly equally divided categorical variables revealed a high intake of bread, moderate total meat and total fish intake and low vegetable and fruit intake were thus associated with a small but significant decrease in the onset risk of dementia, while poultry and cereal were not. These findings are mostly inconsistent with the idea that Mediterranean diet is associated with lower risk of subsequent incident dementia.

## 1. Introduction

Increasing dementia is a matter of public concern. Still, an effective treatment of dementia is not available, so whether dietary and nutrition and dietary patterns are associated with a reduction of aging-related cognitive abilities and the development of dementia is an interesting research question. Previously, numerous prospective studies investigated the association between dietary patterns and the subsequent risk of dementia. Meta-analyses showed reduced risk of subsequent onset of dementia with greater intake of vegetables and fruits [1], of fish [2], and the Mediterranean diet [3] which were characterized by high intake fish, vegetables, fruit, legumes, nuts, seeds, cereals, and olive oil, moderate consumption of fish, poultry, eggs, cheese, yogurt, wine, and low consumption of meat.

However, there are issues with the previous studies. For example, the sample sizes are small (*N* < 10,000) [3]. In addition, in recent times, the associations between nutrition and dementia risk might be affected by multiple reasons such as the development of medicine (e.g., treatment of stroke might lessen the associations between cardiovascular risk and dementia). Related to this, recent big sample size studies showed an association between high body mass index (BMI) and reduced risk of dementia (which is not explained by reverse causation) [4,5] in middle and old age, contrary to the conclusions in middle age suggested by meta-analyses of older studies [6]. Further, some previous studies have methodological problems such as inclusion of dementia a few years after baseline which may lead to concerns toward reverse causations, given that patients with some types of dementia might prefer sugar and carbohydrates and show hyperphagia [7,8], or little consideration to the possible memory function alterations in subjects who will develop dementia. Finally, while the Mediterranean diet was associated with a lower subsequent onset of dementia [3], how each component among the wide range of foods that characterize Mediterranean diet is associated with the risk of dementia is mostly not known. 

Thus, to address these issues, we examined the association of the consumption of basic food categories and the risk of incident dementia >5 years after baseline (to remove the possibility of reverse causations as much as possible) using data from the UK Biobank, which has a follow-up period of about 10 years, around 500,000 participants at baseline and a wide range of individual variables. Our basic hypothesis was that despite the above mentioned issues, foods like those part of the Mediterranean diet would be associated with a reduced risk of dementia. 

## 2. Materials and Methods

### 2.1. Participants

For our study, we used data from the UK Biobank, obtained from a prospective cohort study of a middle-aged population in the United Kingdom and for which the procedures have been described elsewhere (http://www.ukBiobank.ac.uk/wp-content/uploads/2011/11/UK-Biobank-Protocol.pdf, accessed on 5 July 2021). Approval for these experiments was obtained from the North-West Multi-center Research Ethics Committee and written informed consent obtained from each participant. Briefly, participants went to one of 22 assessment centers throughout UK for data collection, with baseline data obtained from 502,505 participants. Our study included the data obtained at the first assessment visit from this cohort (2006–2010). The sample analyzed in each analysis consisted of subjects with all valid independent variables and dependent variables in each analysis.

### 2.2. Assessment of Dietary Intakes

The methods of collection of dietary data in the UK Biobank were previously described and the descriptions in this subsection mostly reproduced from these previous studies [9]. In the assessment center, participants answered 29 questions about their diet using a touch screen. Many of these questions asked about the frequency of consumption of main foods or food groups.

The questions analyzed in this study were those referring to: processed meat, poultry, beef, lamb, pork, oily fish, non-oily fish, fresh fruit, dried fruit, raw vegetables, cooked vegetables, cheese, cereal, tea and coffee. From these items, the following categories of food intake were generated: total meat, total fish, total fruit, total vegetables, cheese, poultry, bread, cereal, tea and coffee as were used previously [9]. For each food group, subjects were divided into four mostly equally distributed groups [9]. More details are provided in the Supplementary Methods.

In addition, the information of the main type of bread consumed (Data ID: 1448: which included “White”, “Brown”, “Wholemeal or wholegrain” and “Other type of bread” as effective answers) and the main type of cereal consumed (Data ID: 1468: which included “Brown cereal”, “Biscuit cereal”, “Oat cereal”, “Muesli” and “other” as effective answers) was obtained and was analyzed as categorical variables as were cases of main analyses.

### 2.3. Sociodemographic and Lifestyle Measurements as Covariates

Self-reported sex was used. From the database, the neighborhood-level socioeconomic status at recruitment (cov1), education level at recruitment (cov2), household income (cov3), current employment status (cov4), metabolic equivalent of task hours (cov5), number of people in household (cov6), height (cov7), BMI (cov8), self-reported health status (cov9), sleep duration (cov10), systolic blood pressure (cov11), current alcohol drinking level (cov12), current tobacco smoking level (cov13), depression score (cov14), race (cov15), diagnosis of diabetes, heart attack, angina, stroke, cancer, overeating, anorexia nervosa, bulimia nervosa, and other serious medical conditions (cov16–24), visuospatial memory task performance (performance worse than 2SD were excluded) (cov25) were extracted or calculated and included as covariates. For additional details, refer to the Supplementary Methods section.

### 2.4. Psychological Data Analyses

Psychological data were analyzed using the Predictive Analysis Software, version 22.0.0 (SPSS Inc., Chicago, IL, USA; 2010). Cox proportional hazards models were used to examine the relationships between dietary habits and dementia of all causes, as previously described [10]. All-cause dementia was determined using hospital inpatient records and links to death register data. For more details, see the Supplementary Methods. The following subjects were removed from the analyses: (a) subjects already diagnosed with dementia at baseline, (b) those diagnosed with dementia or those who died within five years after baseline, (c) self-reported dementia or cognitive impairment at baseline, (d) self-reported dementia without a diagnosis in either hospital inpatient records or death register data, and (e) those with visuospatial memory performance lower than 2SD. The time scale considered spanned from the time of the first assessment visit and until the 28 February 2018. For each analysis, sex, age at baseline, and cov1–cov25 values were used as covariates. 

Results with a *p* < 0.05, corrected for false-discovery rates (FDRs) using the two-stage sharpened method [11] in analyses of group differences of each food type, were considered statistically significant. This correction was applied to *p* values of the analyses of group differences for each type of food intake. Note the lower *p* values in FDR analyses (compared with uncorrected *p* values) of two-stage sharpened methods occur when there are strong signals (lower *p* values) in other analyses [12].

We also reported the supplemental analyses of type of bread consumed and type of cereal consumed. 

## 3. Results

### 3.1. Basic Baseline Data

Baseline psychological data for all participants is provided in Table 1.

### 3.2. Prospective Analysis of Dementia

**Figure 1:**
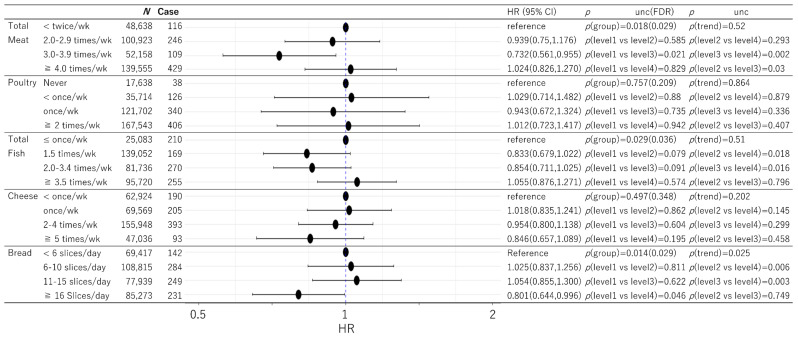
Statistical values and hazard ratios (95% CIs) for the associations between total meat, poultry, total fish, cheese, and bread intake and incident dementia >5 years after baseline in the UK Biobank data. Participants are categorized according to their intake level at baseline. “P(group)” indicates the *p* values of the existence of the group difference among all the groups. Note the lower *p* values in FDR analyses (compared with uncorrected *p* values) of two-stage sharpened methods occur when there are strong signals (lower *p* values) in other analyses [12]. 95% CI = 95% confidence interval. HR = hazard ratio. FDR = False discovery rate.

**Figure 2:**
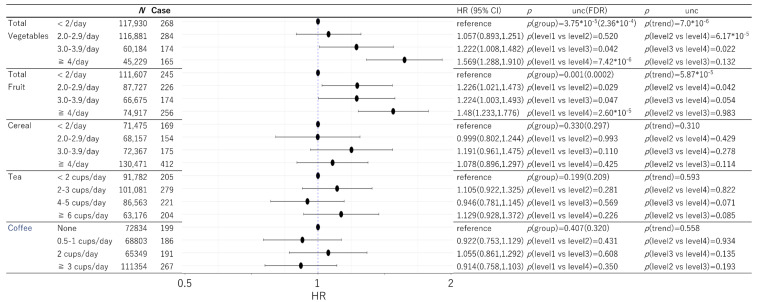
Statistical values and hazard ratios (95% CIs) for the associations between total vegetables, total fruit, cereal, tea, and coffee intake and incident dementia >5 years after baseline in UK Biobank data. Participants are categorized according to their intake level at baseline. “p(group)” indicates the *p* values of the existence of the group difference among all the groups. Note the lower *p* values in FDR analyses (compared with uncorrected *p* values) of two-stage sharpened methods occur when there are strong signals (lower *p* values) in other analyses [12]. 95% CI = 95% confidence interval. HR = hazard ratio. FDR = False discovery rate.

Among the data of 502,505 participants in the present project, 43 participants had only self-reported dementia and among the remaining participants, 182 had dementia records diagnosed before baseline. Among the rest, 755 participants had dementia records diagnosed within 5 years after baseline and 8462 participants had died for other reasons within this time. The number of participants and cases included in the analyses after excluding those with missing data for any one variable in the analysis are shown in Figure 1 and Figure 2.

Cox proportional hazard models dividing subjects into four groups based on the intake of each food category (levels 1 to 4) were determined after adjusting for potential confounding variables and correction for multiple comparisons. Total meat, total fish, bread, total vegetables, and total fruit intake showed significant group differences, while poultry, cheese, cereal, tea and coffee did not. 

In the case of total meat, the group of the second highest intake (level 3) showed significantly lower risk of dementia compared with each of the other 3 groups (Figure 1). 

In the case of total fish, the group with the highest intake (level 4) showed higher risk of dementia compared with the second and third highest intake groups (significance in post-hoc comparisons between level 2 < level 4 and level 3 < level 4). As previous studies comparing no fish intake with other groups showed a steep reduction of health risks with low fish intake [2], but without a dose-response relationship beyond such levels [13], we further conducted modified supplemental analyses dividing level 1 (≤1 times/week) into level 0 (no intake of fish) and level 1′ (0 < x ≤ 1 times/week) (to a total of five intake levels in this supplemental analysis). In this analysis, the no intake group (level 0) showed the highest risk, while a significant reduction in risk of dementia was observed for intake level 1′(HR: 0.688, 95% CI: 0.479–0.988, *p* = 0.043), intake level 2 (HR: 0.620, 95% CI: 0.435–0.882, *p* = 0.008), and intake level 3 (HR: 0.436, 95% CI: 0.250–0.760, *p* = 0.003), but not for intake level 4 (HR: 0.765, 95% CI: 0.537–1.091, *p* = 0.140). This supplemental analysis may suggest that compared with no fish intake, moderate fish intake is also associated with a reduction of the subsequent risk of incident dementia. 

In the case of bread, the highest intake group showed a significantly lower risk of dementia compared with each of the other 3 groups (Figure 1).

In the case of vegetables and fruit, relatively clear dose-response patterns were observed and, in both of the cases, the highest intake group showed the highest risk of dementia while that with the lowest intake showed the lowest risk and of the middle intake groups (levels 2 and 3) had middle risk (Figure 2).

Statistical values and HR were presented in Figure 1 and Figure 2. 

In addition, we analyzed the associations between the main type of bread intake and the main type of cereal intake and incident dementia >5 years after baseline. No significant associations were observed. Estimated fibre content/portion in each type of main bread and cereal intake was also not associated with the observed hazard risk of incident dementia. Statistical values and HR are presented in Table 2. 

In addition, considering the average age of the present cohort (56.5) is relatively younger compared with the age at which dementia frequently occurs, we also repeated analyses using the sample with the age ≥60. The results of comparisons of statistical values of two types are presented in the Supplementary Table S1. Overall, although *p* values of significant group differences tended to be weaker in this subsample analysis, all significant results in the main text, remained significant at the *p* value (uncorrected) level, and these results did not alter our conclusions. 

## 4. Discussion

Our study employed a large data sample to evaluate the associations between dietary patterns and subsequent onset of dementia, correcting for a wide range of potential confounders and removing onset of dementia within 5 years after baseline. Partly consistent with our hypothesis, the moderate fish intake level was associated with significantly lower risk of dementia. However, most of our results were not congruent with our hypothesis and the notion that elements of the Mediterranean diet are associated with a reduction of risk of dementia; on the contrary, our results revealed that a moderately high meat intake, high bread intake and low intake of vegetables and fruit were associated with lower risk of subsequent onset of dementia. In any event, this is a prospective observational study and not indicative of causality. The present results were obtained after correcting for the effects of physical activity levels, BMI and a wide range of diseases, and health status, and removing onset within 5 years after baseline and subjects with low memory performance at baseline. 

In this study, moderately high meat intake was associated with a reduced risk of subsequent onset of dementia compared with the highest and lowest meat intake. Previous evidence has suggested a positive association between meat intake and cardiovascular diseases and stroke [15]. Further, this finding is incongruent with the previously reported notion that the Mediterranean diet is associated with a reduced risk of incident dementia, since one of the characteristics of the Mediterranean diet is eating less meat [16]. A recent study reported similar findings in a much smaller sample (*N* = 5934), showing that low meat consumption (≤once/week) was associated with an increased risk of dementia compared with regular consumption (≥4 times/week). Further, a previous cohort study revealed that an adequate protein (20% of total calorie intake) and high fat intake were associated with a significant reduction of dementia risk [17]. In addition, a previous study showed that among 5691 elderly people, a lower meat intake was associated with cognitive decline [18]. The present study may be consistent with these studies. The reason why moderate meat or protein intake is associated with a reduced risk of dementia may be because it is relevant to maintain the integrity of neuronal membranes and brain cells, and because some amino acids are critical as precursors in the biosynthesis of neurotransmitters [15]. Further, it is known protein intake helps muscle retention in aging adults, which may be important for dementia prevention [15]. On the other hand, an excess intake may not be associated with reduced risk of dementia partly because of the increased risk of cardiovascular diseases and stroke. However, these are pure speculations and future studies are needed to investigate these issues. 

In this study, moderately high total fish intake was associated with reduced risk of subsequent onset of dementia. Previous accumulating evidence has suggested that compared with no fish intake, fish intake was associated with reduced risk of dementia, although whether dose-response relationships exist differs depending on the type of analysis [2,19]. Moreover, higher fish intake is associated with a lower risk of cardiovascular disease death but a dose-response relationship beyond a modest intake level has not been observed [12]. Among the nutrients in fish, docosahexaenoic acid (DHA) and eicosapentaenoic (EPA) are considered to have protective effects against neurodegeneration [2]. On the other hand, the reason why the group with the highest intake showed relatively large risks of dementia can be only speculated. Since fish have methyl-mercury, which is a neurotoxin, and increased fish intake has been shown to be associated with mercury levels in the brain [20], this may be a potential cause for the results of the present study. Through this mechanism, the present study might find the adverse effect of too high intake due to the large sample size, which was the level not seen in the previous studies. 

Higher fruit and vegetable intake was associated with an increased risk of subsequent onset of dementia. The present finding is apparently in contradiction to a recent meta-analysis that reported significant associations between higher intake of fruit and vegetables and reduced risk of dementia [1]. However, this finding may be similar to a recent controversy on the association between greater BMI and reduced risk of dementia. Meta-analyses of old studies favored the associations of lower BMI and reduced risk of dementia in middle age [6], but recent multiple mega-sample studies including data from the UK Biobank consistently confirmed the association between higher BMI and reduced risk of dementia in middle and old age [4,5]. The reason for the association between higher intake of vegetables and fruit and dementia risk may be due to the fact that a strong preference for such foods may reflect a relatively low intake of protein, lipid and late-life cholesterol, all thought to be protective against dementia [4]. However, the reasons behind the discrepancy between the present finding and previous meta-analysis are not clear. We speculate that proper design control (removal of onset within 5 years after baseline, corrections for confounders such as education), modern development of drugs to treat stroke and infarction, diabetes (which may not strongly lead to risk of dementia as previously considered), as well as prevalence of vitamins or other essential nutrients provided by vegetables and fruit, might contribute to the differences. Future studies are therefore needed to investigate the rationale for this discrepancy. 

We compared changes in the hazard risk of dementia associated with dietary habits with other factors in this study. For example, in the analysis of total meat intake, we found that a 1-point increase in household income level was associated with a hazard risk of 0.889 (95%CI:0.818–0.962, *p* = 0.004) (in this study, the income level was as follows (1) < £18,000, (2) £18,000 to £30,999, (3) £31,000 to £51,999, (4) £52,000 to £100,000, and (5) > £100,000). Having a baseline diagnosis of diabetes and stroke also increased the hazard risk of dementia to 1.649 (95%CI:1.342–2.026, *p* = 2.0 × 10^−6^) and 1.997 (95%CI:1.520–2.624, *p* = 2.0 × 10^−6^), respectively. On the other hand, compared to the group with the lowest intake levels of fruits and vegetables, the group with the highest levels of fruits and vegetables had a hazard risk of 1.480 (95%CI:1.233–1.776) and 1.569 (95%CI:1.288–1.910), respectively. The effect sizes of these dietary differences were similar to the effect size of diagnosis of diabetes, corresponding to the effect size of a four-level decrease in household income. The optimal level of total meat intake compared with the lowest intake level, the optimal level of fish intake compared with the highest intake level, and the highest bread intake level compared with the lowest intake level were associated with hazard risks of 0.732 (95%CI:0.561–0.955), 0.789 (95%CI:0.649–0.960) and 0.801 (95%CI:0.644–0.996), respectively. These changes are not as high as the risk associated with diagnosis of diabetes and correspond to a change of approximately a two-level increase in household income.

In this study, the highest bread intake level was associated with a significantly reduced risk of dementia. The association between bread and risk of dementia was not frequently reported. A small study (*N* < 1000) reported that subjects with higher caloric input from carbohydrates showed a higher risk of dementia, which may not be partly consistent with the present findings [17]. Further, a previous study showed that subjects with certain types of dementia prefer to ingest sugar and carbohydrates [8]. The mechanisms behind the present associations are not clear, however, and they may be related to the above-described association between higher BMI and lower risk of dementia. Future studies need to reveal the mechanisms behind the present observation. 

We do not have enough data to conclude whether a particular type of dietary pattern is closely associated with the risk of dementia. Dietary habits that conflict with high bread intake, moderate meat and fish intake, and low vegetable and fruit intake appear to be similar to vegetarian diets, but our data do not allow for a similar analysis of the association with dementia risk with respect to high and low intake of legumes and dairy products that are necessary to characterize a vegetarian diet. Similarly, to simply characterize the above dietary patterns as a low-calorie diet or low protein diet, there is a discrepancy in the results of the fruit and bread analysis.

This study has some limitations. First, this study is an observational prospective study and not an interventional study. Although we corrected for a wide range of potential confounding factors and removed subjects diagnosed with dementia within 5 years after baseline, other uncorrected factors might affect our findings. Alternatively, subjects with certain risks of dementia may tend to follow certain dietary habits. To evaluate the possibility that subjects with health risks tend to eat more vegetables and fruit, and less meat and bread, we evaluated the overall health rating at each food consumption level. The results in Supplementary Table S2 show that subjects with the dietary patterns of increased risk of dementia did not show overall low health, while low overall health status was robustly associated with increased risk of incident dementia in the main analyses. In fact, as for the total fish, bread, total vegetables and fruit, the group with the highest risk of incident dementia showed an overall high health status. However, these issues could ultimately be solved by well-designed intervention studies. In addition, despite removing subjects with dementia onset within 5 years after baseline from analyses, changes to biomarkers of Alzheimer’s disease begin >20 years before onset, and the cognitive changes of people who will develop Alzheimer’s disease begin 10 years before onset [21], so it is quite possible that some dietary behaviors of the participants who developed dementia within the observation period of this study were affected by the neurological processes of pre-clinical condition of dementia, instead of the opposite. Therefore, the present findings should be considered as a representation of the diet characteristics of people who will develop dementia. These are the limitations most of the longitudinal observational studies have and future intervention studies must demonstrate the causality. Also, in the dementia data from the UK Biobank, the first time participants were on average 56.5 years old and had an average follow-up of approximately 10 years. Although representative studies in many fields have been conducted using this database, this age group is slightly before the age at which dementia frequently occurs. This may have somewhat reduced the statistical power of the study. Another limitation of this study is that it does not clarify through what mechanism dietary habits are associated with changes in dementia risk. Brain imaging studies, gut microbiome studies, and epigenetic studies may provide insight into such mechanisms, but longitudinal brain imaging data are not sufficiently accumulated in the UK Biobank to statistically detect the effect size of dietary habits. For the latter two, the UK Biobank does not seem to have adequate longitudinal data to evaluate effects of dietary habits, as far as we are aware. Future studies should be conducted through these methods to elucidate the mechanisms of observed associations in detail.

## 5. Conclusions

Previously, several prospective observation studies investigated the associations between dietary characteristics and subsequent onset of dementia. The present study employed a large data sample from the UK Biobank, removed patients with onset of dementia within 5 years after baseline and those with particularly low memory performance at baseline, and corrected for a wide range of potential confounders. Our results demonstrate that moderate total meat and total fish intake, low vegetable and fruit intake, and high bread intake characterize people who are less likely to later develop dementia. These results are mostly inconsistent with some of the previous findings and the theory that the Mediterranean diet is associated with the reduced risk of dementia. Causality should be confirmed by future investigational studies. If the associations found in this study are confirmed to be causal and critical nutritional components to alter the dementia risk are revealed, it may be possible to apply nutritional supplements and nutritional guidance to correct the dietary patterns that lead to the risk of dementia, and such interventions may reduce the risk of dementia. Future studies need to investigate these issues.

## Figures and Tables

**Table 1:** Baseline characteristics of UK Biobank participants included in the present project (*n* = 502,505).

Item	No. (%)	Mean (SD)	Range
Sex			
Female	273,382 (54.4)		
Male	229,122 (45.6)		
Missing	1		
Age, years		56.5 (8.0)	37–73
Missing	1 (0.0)		
BMI		27.4 (4.8)	12–75
Underweight (18.5 ≥ BMI)	2626 (0.5)		
Normal (25 ≥ BMI > 18.5)	162,523 (32.3)		
Overweight (30 ≥ BMI > 25)	212,097 (42.2)		
Obesity (30 > BMI)	122,153 (24.3)		
Missing	3107 (0.6)		
Average total household income before tax			
< £18,000	97,198 (19.3)		
£18,000 to £30,999	108,177 (21.5)		
£31,000 to £51,999	110,772 (22.0)		
£52,000 to £100,000	86,266 (17.2)		
>£100,000	22,929 (4.6)		
Missing	77,164 (15.4)		
Townsend index of material deprivation		−1.3 (3.1)	−6.25–11.00
Missing	624 (0.1)		
Employment status			
In paid employment or self-employed	287,149 (57.1)		
Not in paid employment or self-employed	212,404 (42.3)		
Missing	2952 (0.6)		
Highest education qualification (years)		13.95 (5.1)	7–20
Missing	10,113 (2.0)		

**Table 2:** Statistical values and hazard ratios (95% CIs) for the associations between the main type of bread intake and the main type of cereal intake and incident dementia >5 years after baseline in UK Biobank data.

	Estimated Fibre Content/Portion (g) [14]	*p* Value	HR (95% CI)
Main type of bread			
white	0.68	*p*(group difference) = 0.596	reference
brown	1.26	*p*(type1 vs. type2) = 0.473	1.082(0.873,1.341)
wholemeal or wholegrain	1.8	*p*(type1 vs. type3) = 0.491	0.946(0.807,1.109)
other type	1.25	*p*(type1 vs. type4) = 0.751	0.943(0.658,1.353)
Main type of cereal			
Bran cereal	7.16	*p*(group difference) = 0.928	reference
Biscuit cereal	2.92	*p*(type1 vs. type2) = 0.945	1.008(0.792,1.285)
Oat cereal	1.92	*p*(type1 vs. type3) = 0.980	0.997(0.797,1.247)
Muesli	4.18	*p*(type1 vs. type4) = 0.637	1.060(0.832,1.351)
Other (e.g., cornflakes, frosties)	0.54	*p*(type1 vs. type5) = 0.663	0.947(0.741,1.210)

95% CI = 95% confidence interval. HR = hazard ratio.

## Data Availability

Data used in this study is accessible by request to the UK Biobank.

## Supplementary Materials

The following are available online at https://www.mdpi.com/article/10.3390/nu13124500/s1, Table S1: Comparisons of results between the analyses using the entire age group and those using the subjects with 60≤ years old, Table S2: Adjusted overall health rating of each dietary intake level and 95% CI for all food groups.
