# Supplementary material for: Diet and Dementia: A Prospective Study
Source: Nutrients. 2021 Dec 16;13(12):4500. doi: 10.3390/nu13124500 (PMC8705494; doi:10.3390/nu13124500)
Supplement: Supplementary file 1 [file nutrients-13-04500-s001.zip › nutrients-1489880-supplementary.pdf]

Supplementary Methods

Additional Information on Study Subjects

Adults between 40 and 69 years old who were registered with the National Health Service (NHS) and lived within 25 miles of the study's evaluation sites were invited by email to take part in the UK Biobank. No exclusion criteria were applied for recruitment.

Dietary Variables

In the conversion of dietary processes, we basically followed a representative study using this data [1], with the descriptions of these processes mostly reproduced from it. For each food category of analysis, subjects were divided into four groups according to their food intake. Cut-off points for each food category were set as whole integers or whole integers plus 0.5, so that all groups had a number of subjects as similar as possible as far as the data distribution allowed [1].

Meat and Fish

Subjects were asked how often they consumed oily fish, non-oily fish, processed meats, poultry, beef, lamb, and pork. The options were "never," "less than once a week," "once a week," "2–4 times a week," "5–6 times a week," "once or more daily', 'do not know," "prefer not to answer." We converted these answers as previously reported [9]. After removing participants with answers of "do not know" and "prefer not to answer," for processed meat, poultry, oily fish and non-oily fish, we combined the top three frequency choices and divided the subjects into four groups: never, <1.0 time per week, 1.0 time per week, and ≥2.0 times per week. To sum the multiple categories of each meat and fish type, first we converted the answers of each in the following way: "Never" = 0, "Less than once a week" = 0.5, "Once a week" = 1, "2–4 times a week" = 3, "5–6 times a week" = 5.5, "Once or more daily" = 7. Then we summed the frequency values of beef, pork, and lamb/mutton to create the frequency of red meat intake. For red meat, we then divided the subjects into four groups: <1 time per week, 1.0–1.9 times per week, 2.0–2.9 times per week, and ≥3.0 times per week. We also summed the frequency values of beef, pork, lamb/mutton and processed meat to create the frequency intake of red and processed intake. For red and processed meat, we then divided the subjects into four groups: <2.0 times per week, 2.0–2.9 times per week, 3.0–3.9 times per week, and ≥4.0 times per week. We also summed the frequency value of oily fish, and non-oily fish to create the total fish intake frequency. For total fish intake, we then divided the subjects into four groups: <1.0 time per week, 1.0–1.9 times per week, 2.0–2.9 times per week, and ≥3.0 times per week.

Bread

With respect to bread, subjects were asked to enter the number of slices of bread they consumed per day, or to choose among the options "less than one," "do not know" or "prefer not to answer." "Less than one" was counted as 0. We divided the subjects into the following four groups, <6.0 slices per day, 6–10 slices per day, 11–15 slices per day, and ≥16 slices per day, which were approximately equally distributed.

Fruit

As for fruit, subjects were asked to enter the number of pieces of fresh fruit and dried fruit each they consumed per day, or to choose among the options "less than one," "do not know" or "prefer not to answer" (examples of what constitute a piece are provided). One piece of fresh fruit was converted to one serving, while two pieces of dried fruit equaled one serving ("Less than one" was counted as 0 in each fruit type). We divided subjects into the following four groups,

<2.0 servings per day, 2.0–2.9 servings per day, 3.0–3.9 servings per day, and ≥4.0 servings per day, which were approximately equally distributed.

Vegetables

In terms of vegetables, subjects were asked to enter the number of heaped tablespoons of every cooked and salad/raw vegetable they consumed per day, or to choose "less than one," "do not know," or "prefer not to answer." As in the previous study, two combined heaped tablespoons were counted as one serving (<1 was counted as 0). Next, we divided the subjects into the following four groups, <2.0 servings per day, 2.0–2.9 servings per day, 3.0–3.9 servings per day, and ≥4.0 servings per day, which were approximately equally distributed.

Cereal

For cereal, subjects were asked to enter the amount of bowls of cereal they consumed per day, or to choose "less than one," "do not know" or "prefer not to answer." "Less than one" is counted as 0. We divided subjects into the following four groups, <2 bowls per day, 2–4 bowls per day, 5–6 bowls per day, and ≥7.0 bowls per day, which were approximately equally distributed.

Cheese

Subjects were asked how often they consume cheese. The options were "never," "less than once a week," "once a week," "2–4 times a week," "5–6 times a week," "once or more daily," "do not know," and "prefer not to answer." As handled previously [9], after removing the participants with answers of "do not know" and "prefer not to answer," we combined the top two and bottom two frequency choices and divided the subjects into four groups <1.0 time per week, 1.0 time per week, 2.0–4.9 times per week, and ≥5.0 times per week.

Tea and Coffee

Subjects were asked to enter the number of cups of tea, including black and green tea, and coffee (including decaffeinated coffee) they consume per day, or choose "less than one," "do not know" or "prefer not to answer," <1 was counted as 0. As in the previous study [9], in the case of tea, we divided subjects into the following four groups, <2.0 servings per day, <2.0 cups per day, 2.0–3.9 cups per day, 4.0–5.9 cups per day, and ≥6.0 cups per day. In the case of coffee, we divided subjects into the following four groups, 0 cups/day, 0.5–1.9 cups per day, 2.0–2.9 cups per day, and ≥3.0 cups per day.

Details on Sociodemographic and Lifestyle Measures

(cov1) Neighborhood-level socioeconomic status was measured by the Townsend index of material deprivation [2]. Status was calculated based on the postcode of the subject's address and represented a composite index of four postcode-level socioeconomic status variables: household overcrowding, unemployment, non-home ownership, and non-car ownership. A higher score implied a lower socioeconomic status. For this variable, only the value at recruitment was available and was used for all analyses.

(cov2) The education level was based on self-reported data. Education level categories of participant choices were transformed into numerical values based on a previous study [3] as follows: "College or University degree" = 20 years; "A levels/AS levels or equivalent" = 13 years; "O levels/GCSEs or equivalent" = 10 years; "CSEs or equivalent" = 10 years; "NVQ or HND or HNC or equivalent" = 19 years; "Other professional qualifications e.g., nursing, teaching" = 15 years; "None of the above" = 7 years; "Prefer not to answer" = missing. For this variable, only the value at recruitment was available so it was used for all analyses.

(cov3) The household income was the self-reported total income (before taxes) received by the subject's household. The available choices were <£18,000, £18,000 to £30,999, £100,000, £31,000 to £51,999, £52,000 to £100,000, >£100,000, do not know, and prefer not to answer. We converted these choices into ordinal variables between 1 and 5 (>£100,000 = 5) [4] after excluding the answers of "do not know" and "prefer not to answer," as elsewhere.

(cov4) The variable "current employment status" was used to describe the participants' occupation. The responses to the variable were: "In paid employment or self-employed," "Retired," "Looking after home and/or family," "Unable to work because of sickness or disability," "Unemployed," "Doing unpaid or voluntary work," "Full or part-time student," and "None of the above." Multiple responses were allowed. Subject responses were classified as either "In paid employment or self-employed" or not.

(cov5) Physical activity level was calculated from the recorded items from the International Physical Activity Questionnaire short form and converted into a single measure of total physical activity in the metabolic equivalent of task hours (MET). For more details, see [5].

(cov6) Participants were asked about the number of people in their household (including institutions such as care homes). Answers were assigned one of four variables: 1 (single person), 2 (two people), 3 (three people), and 4 (≥4 people) as elsewhere [6].

(cov7,8) Body weight was measured using Tanita BC418MA scales. Height was measured using a Seca height measure. BMI was calculated from the measured height and weight.

(cov9) Participants were asked about their health status, with possible answers being excellent, good, fair, poor and converted to values of 4, 3, 2, and 1, respectively, before input in statistical analyses.

(cov10) Sleep duration was assessed with the item "About how many hours sleep do you get in every 24 h? (Please include naps.)" Responses were coded as integers and divided into 4 groups, (a) ≤4 h, (b) 5 h or 6 h, (c) 7 h or 8 h, (d) ≤9 h.

(cov11) Systolic blood pressure was measured using a digital BP monitor (Omron), or a manual sphygmomanometer when the digital monitor was not available. One or two readings were taken and we used the average, as described in a previous study [7].

(cov12) The current alcohol intake level was calculated as previously described [8]. Study participants were asked to describe their current drinking status (never, previous, current, prefer not to say), and estimate their current alcohol intake frequency (daily or almost daily, three or four times a week, once or twice a week, one to three times a month, special occasions only, never, prefer not to say). Individuals reporting a current intake frequency of ≥1–2 a week were asked to estimate their average weekly intake of a range of different alcoholic beverages (red wine, white wine, champagne, beer, cider, spirits, fortified wine). From these variables, we calculated an average intake of alcoholic units per week. This was derived by combining the self-reported estimated intake of the different alcoholic beverages across all types. This calculation used the following measurement units for each of the five alcoholic drink types: measures for spirits, glasses for wines and pints for beer/cider, were estimated to be equivalent to 1, 2 and 2.5 units respectively. Individuals reporting current intake frequency of "one to three times a month," "special occasions only," or "never," were assumed to have a weekly alcohol consumption volume of 0. Based on the obtained variable, four categories (a) 0, (b) $0 < x \leq 14$, (c) $14 < x < 28$ and (d) $28 < x$ were generated.

(cov13) Participants were asked about their current tobacco smoking status. Possible answers were 1 (No), 2 (Only occasionally), and 3 (Yes, on most or all days) and treated as a categorical variable. Responses of "prefer not to answer" were excluded.

(cov14) Depressive symptoms were measured by the 4-item Patient Health Questionnaire-4 (PHQ-4) [9], which was administered at all four patients visits to assessment centers. This measurement has an area under the curve of 0.79 for its correlation with a depression diagnosis [10]. For other information on the reliability and validity of this measurement technique, see [10].

(cov15) Ethnicity was self-reported, and possible effective answers divided into white or other, and analyzed.
(cov16–24) Participants were asked about the existence of a medical diagnosis of diabetes, heart attack, angina, stroke, cancer, overeating, anorexia nervosa, bulimia nervosa, and other serious medical conditions (item ID: 1049, 6150, 2453 2473, 20544). A dichotomized variable of existence for each condition was generated based on this answer.
(cov25) Visuospatial memory was measured by the "pairs-matching" task. In this test, participants were asked to memorize the positions of six card pairs, and then match them from memory while making as few errors as possible. Scores on the pairs-matching test corresponded to the number of errors that participants made and therefore, higher scores reflected poorer cognitive functions.

Determination of Dementia

For the determination of dementia of all causes, we followed methods established in a previous study [11], with descriptions in this subsection mostly being reproduced from this study. All-cause dementia was determined based on hospital inpatient records containing data on admissions and diagnoses from the Hospital Episode Statistics for England, Scottish Morbidity Record data for Scotland, and the Patient Episode Database for Wales. Additional cases were identified through death register data provided by the NHS Digital for England and Wales and the Information and Statistics Division for Scotland. Diagnoses were recorded using the International Classification of Diseases (ICD) coding system. Participants with dementia were identified as having a primary/secondary diagnosis (hospital records) or underlying/contributory cause of death (death register) using ICD-9 and ICD-10 codes for Alzheimer disease and other dementia classifications.

Supplementary Table S1. Comparisons of results between the analyses using the entire age group and those using the subjects with 60≤ years old.

			**Analyses Using the Entire Age Group**		**Analyses Using the Sample with the Age ≧ 60**	
	**Level**	**Amount**	**HR (95% CI)**	***p* Value**	**HR (95% CI)**	***p* Value**
Total meat	1	<twice/wk	reference	p(group difference) = 0.018	reference	p(group difference) = 0.011
	2	2.0–2.9 times/wk	0.939(0.75,1.176)	p(level1 vs. level2) = 0.585	0.88(0.686,1.128)	p(level1 vs. level2) = 0.312
	3	3.0–3.9 times/wk	0.732(0.561,0.955)	p(level1 vs. level3) = 0.021	0.69(0.515,0.925)	p(level1 vs. level3) = 0.013
	4	≥4.0 times/day	1.024(0.826,1.27)	p(level1 vs. level4) = 0.829	1.002(0.791,1.269)	p(level1 vs. level4) = 0.989
Poultry	1	never	reference	p(group difference) = 0.757	reference	p(group difference) = 0.778
	2	<once/wk	1.029(0.714,1.482)	p(level1 vs. level2) = 0.88	0.945(0.636,1.404)	p(level1 vs. level2) = 0.779
	3	once/wk	0.943(0.672,1.324)	p(level1 vs. level3) = 0.735	0.863(0.597,1.249)	p(level1 vs. level3) = 0.435
	4	≥2 times/day	1.012(0.723,1.417)	p(level1 vs. level4) = 0.942	0.903(0.626,1.304)	p(level1 vs. level4) = 0.586
Total fish	1	≤ once/wk	reference	p(group difference) = 0.029	reference	p(group difference) = 0.039
	2	1.5 times/wk	0.833(0.679,1.022)	p(level1 vs. level2) = 0.079	0.843(0.672,1.058)	p(level1 vs. level2) = 0.141
	3	2.0–3.4 times/wk	0.854(0.711,1.025)	p(level1 vs. level3) = 0.091	0.842(0.686,1.032)	p(level1 vs. level3) = 0.097
	4	≥3.5 times/day	1.055(0.876,1.271)	p(level1 vs. level4) = 0.574	1.062(0.865,1.304)	p(level1 vs. level4) = 0.562
Cheese	1	<once/wk	reference	p(group difference) = 0.497	reference	p(group difference) = 0.496
	2	once/wk	1.018(0.835,1.241)	p(level1 vs. level2) = 0.862	0.956(0.771,1.185)	p(level1 vs. level2) = 0.68
	3	2–4 times/wk	0.954(0.8,1.138)	p(level1 vs. level3) = 0.604	0.88(0.727,1.064)	p(level1 vs. level3) = 0.188
	4	≥5 times/day	0.846(0.657,1.089)	p(level1 vs. level4) = 0.195	0.853(0.65,1.119)	p(level1 vs. level4) = 0.252
Bread	1	<6 slices/day	reference	p(group difference) = 0.014	reference	p(group difference) = 0.05
	2	6–10 slices/day	1.025(0.837,1.256)	p(level1 vs. level2) = 0.811	1.079(0.858,1.357)	p(level1 vs. level2) = 0.517
	3	11–15 slices/day	1.054(0.855,1.3)	p(level1 vs. level3) = 0.622	1.107(0.876,1.4)	p(level1 vs. level3) = 0.395

	4	≥16 bowls/day	0.801(0.644,0.996)	p(level1 vs. level4) = 0.046	0.859(0.674,1.095)	p(level1 vs. level4) = 0.22
Total vegetables	1	<2/day	reference	p(group difference) = 3.75×10^{-5}	reference	p(group difference) = $1.19*10^{-4}$
	2	2.0–2.9/day	1.057(0.893,1.251)	p(level1 vs. level2) = 0.52	1.01(0.839,1.215)	p(level1 vs. level2) = 0.919
	3	3.0–3.9/day	1.222(1.008,1.482)	p(level1 vs. level3) = 0.042	1.204(0.977,1.485)	p(level1 vs. level3) = 0.082
	4	≥4/day	1.569(1.288,1.91)	p(level1 vs. level4) = 7.42×10^{-6}	1.557(1.256,1.93)	p(level1 vs. level4) = $5.31*10^{-5}$
Total fruit	1	<2/day	reference	p(group difference) = 0.001	reference	p(group difference) = 0.002
	2	2.0–2.9/day	1.226(1.021,1.473)	p(level1 vs. level2) = 0.029	1.28(1.046,1.566)	p(level1 vs. level2) = 0.017
	3	3.0–3.9/day	1.224(1.003,1.493)	p(level1 vs. level3) = 0.047	1.299(1.045,1.613)	p(level1 vs. level3) = 0.018
	4	≥4/day	1.48(1.233,1.776)	p(level1 vs. level4) = 2.60×10^{-5}	1.486(1.214,1.818)	p(level1 vs. level4) = $1.19*10^{-4}$
Cereal	1	<2 bowls/day	reference	p(group difference) = 0.33	reference	p(group difference) = 0.665
	2	2–4 bowls/day	0.999(0.802,1.244)	p(level1 vs. level2) = 0.993	0.898(0.705,1.144)	p(level1 vs. level2) = 0.382
	3	5–6 bowls/day	1.191(0.961,1.475)	p(level1 vs. level3) = 0.11	1.038(0.819,1.316)	p(level1 vs. level3) = 0.758
	4	≥7 bowls/day	1.078(0.896,1.297)	p(level1 vs. level4) = 0.425	1.01(0.828,1.231)	p(level1 vs. level4) = 0.925
Tea	1	<2 cups/day	reference	p(group difference) = 0.199	reference	p(group difference) = 0.124
	2	2–3 cups/day	1.105(0.922,1.325)	p(level1 vs. level2) = 0.281	1.179(0.965,1.441)	p(level1 vs. level2) = 0.107
	3	4–5 cups/day	0.946(0.781,1.145)	p(level1 vs. level3) = 0.569	0.98(0.793,1.212)	p(level1 vs. level3) = 0.854
	4	≥6 cups/day	1.129(0.928,1.372)	p(level1 vs. level4) = 0.226	1.176(0.946,1.461)	p(level1 vs. level4) = 0.145
Coffee	1	None	reference	p(group difference) = 0.407	reference	p(group difference) = 0.445
	2	0.5–1 cups/day	0.922(0.753,1.129)	p(level1 vs. level2) = 0.431	0.866(0.695,1.08)	p(level1 vs. level2) = 0.202
	3	2 cups/day	1.055(0.861,1.292)	p(level1 vs. level3) = 0.608	0.96(0.769,1.199)	p(level1 vs. level3) = 0.721
	4	≥3 cups/day	0.914(0.758,1.103)	p(level1 vs. level4) = 0.35	0.869(0.708,1.067)	p(level1 vs. level4) = 0.181

Supplementary Table S2. Adjusted overall health rating of each dietary intake level and 95% CI for all food groups.

	Level1	Level2	Level3	Level4
Total meat	2.899 (2.893–2.904)	2.919 (2.915–2.923)	2.904 (2.899–2.909)	2.854 (2.851–2.857)
Total fish	2.814 (2.806–2.831)	2.873 (2.870–2.877)	2.906 (2.902–2.910)	2.908 (2.904–2.912)
Bread	2.909 (2.904–2.913)	2.888 (2.884–2.892)	2.885 (2.881–2.890)	2.871 (2.866–2.875)
Total vegetables	2.838 (2.835–2.842)	2.901 (2.898–2.905)	2.925 (2.920–2.930)	2.940 (2.934–2.946)
Total fruit	2.820 (2.816–2.823)	2.894 (2.890–2.898)	2.922 (2.917–2.926)	2.950 (2.946–2.954)

Results of analyses of covariance after adjusting for basic covariates (age, sex, neighborhood-level socioeconomic status, education length, household income, current employment status, BMI, height, and race). Values were covariate adjusted values in ANCOVAs.

References

1. Bradbury, K.E.; Murphy, N.; Key, T.J. Diet and colorectal cancer in UK Biobank: a prospective study. *Int. J. Epidemiol.* **2020**, *49*, 246-258.
2. Townsend, P. Deprivation. *J. Soc. Policy* **1987**, *16*, 125-146.

3. Okbay, A.; Beauchamp, J.P.; Fontana, M.A.; Lee, J.J.; Pers, T.H.; Rietveld, C.A.; Turley, P.; Chen, G.B.; Emilsson, V.; Meddens, S.F., et al. Genome-wide association study identifies 74 loci associated with educational attainment. *Nature* **2016**, *533*, 539-542, doi:10.1038/nature17671.
4. Shen, X.; Cox, S.R.; Adams, M.J.; Howard, D.M.; Lawrie, S.M.; Ritchie, S.J.; Bastin, M.E.; Deary, I.J.; McIntosh, A.M.; Whalley, H.C. Resting-state connectivity and its association with cognitive performance, educational attainment, and household income in the UK Biobank. *Biological Psychiatry: Cognitive Neuroscience and Neuroimaging* **2018**, *3*, 878-886.
5. Cullen, B.; Newby, D.; Lee, D.; Lyall, D.M.; Nevado-Holgado, A.J.; Evans, J.J.; Pell, J.P.; Lovestone, S.; Cavanagh, J. Cross-sectional and longitudinal analyses of outdoor air pollution exposure and cognitive function in UK Biobank. *Scientific reports* **2018**, *8*, 1-14.
6. Sarkar, S.N.; Huang, R.-Q.; Logan, S.M.; Yi, K.D.; Dillon, G.H.; Simpkins, J.W. Estrogens directly potentiate neuronal L-type Ca2+ channels. *Proceedings of the National Academy of Sciences* **2008**, *105*, 15148-15153.
7. Veldsman, M.; Kindalova, P.; Husain, M.; Kosmidis, I.; Nichols, T.E. Spatial distribution and cognitive impact of cerebrovascular risk-related white matter hyperintensities. *NeuroImage: Clinical* **2020**, *28*, 102405.
8. Howe, L.J.; Lawson, D.J.; Davies, N.M.; Pourcain, B.S.; Lewis, S.J.; Smith, G.D.; Hemani, G. Genetic evidence for assortative mating on alcohol consumption in the UK Biobank. *Nat. Commun.* **2019**, *10*, 1-10.
9. Batty, G.D.; McIntosh, A.M.; Russ, T.C.; Deary, I.J.; Gale, C.R. Psychological distress, neuroticism, and cause-specific mortality: early prospective evidence from UK Biobank. *J. Epidemiol. Community Health* **2016**, *70*, 1136-1139.
10. Khubchandani, J.; Brey, R.; Kotecki, J.; Kleinfelder, J.; Anderson, J. The psychometric properties of PHQ-4 depression and anxiety screening scale among college students. *Arch. Psychiatr. Nurs.* **2016**, *30*, 457-462.
11. Lourida, I.; Hannon, E.; Littlejohns, T.J.; Langa, K.M.; Hyppönen, E.; Kuźma, E.; Llewellyn, D.J. Association of lifestyle and genetic risk with incidence of dementia. *JAMA* **2019**, *322*, 430-437.
